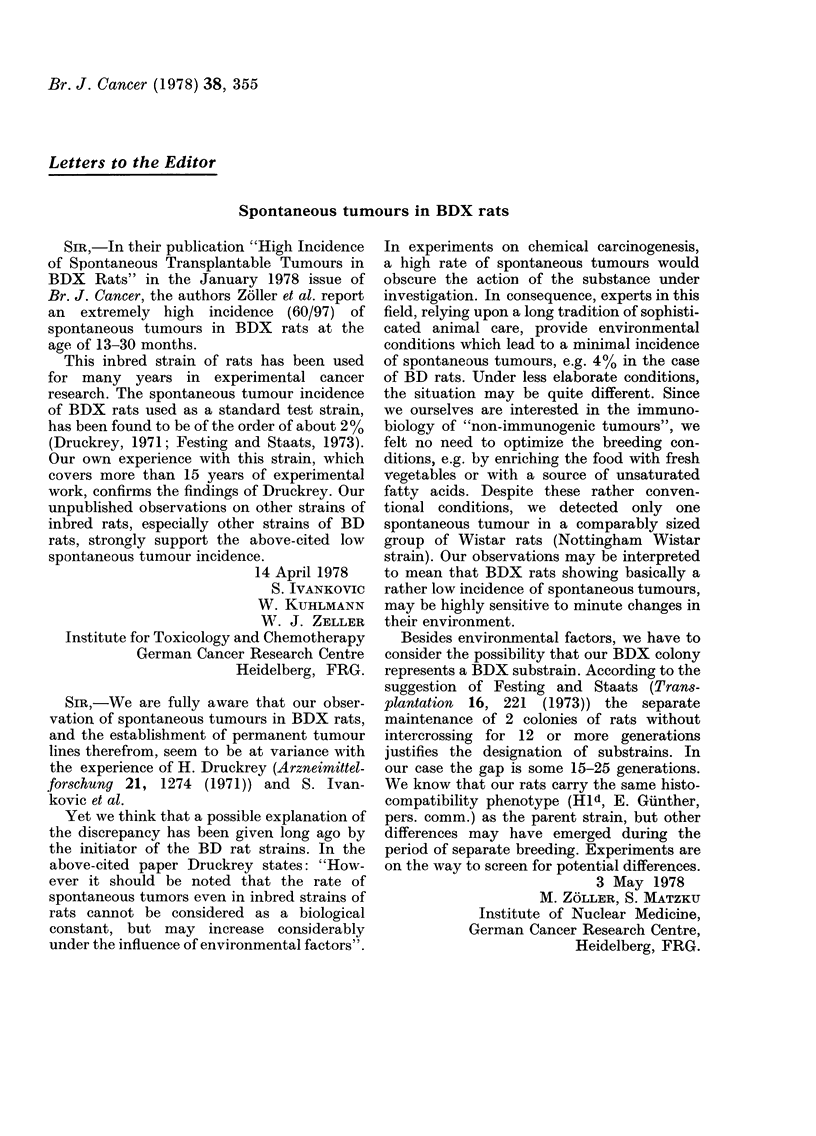# Spontaneous tumours in BDX rats

**Published:** 1978-08

**Authors:** M. Zöller, S. Matzku


### Spontaneous tumours in BDX rats

German Cancer Research Centre

Heidelberg, FRG.

SIR,—We are fully aware that our observation of spontaneous tumours in BDX rats, and the establishment of permanent tumour lines therefrom, seem to be at variance with the experience of H. Druckrey (*Arzneimittelforschung* **21**, 1274 (1971)) and S. Ivankovic *et al.*

Yet we think that a possible explanation of the discrepancy has been given long ago by the initiator of the BD rat strains. In the above-cited paper Druckrey states: "However it should be noted that the rate of spontaneous tumors even in inbred strains of rats cannot be considered as a biological constant, but may increase considerably under the influence of environmental factors". In experiments on chemical carcinogenesis, a high rate of spontaneous tumours would obscure the action of the substance under investigation. In consequence, experts in this field, relying upon a long tradition of sophisticated animal care, provide environmental conditions which lead to a minimal incidence of spontaneous tumours, e.g. 4% in the case of BD rats. Under less elaborate conditions, the situation may be quite different. Since we ourselves are interested in the immunobiology of "non-immunogenic tumours", we felt no need to optimize the breeding conditions, e.g. by enriching the food with fresh vegetables or with a source of unsaturated fatty acids. Despite these rather conventional conditions, we detected only one spontaneous tumour in a comparably sized group of Wistar rats (Nottingham Wistar strain). Our observations may be interpreted to mean that BDX rats showing basically a rather low incidence of spontaneous tumours, may be highly sensitive to minute changes in their environment.

Besides environmental factors, we have to consider the possibility that our BDX colony represents a BDX substrain. According to the suggestion of Festing and Staats (*Transplantation* **16**, 221 (1973)) the separate maintenance of 2 colonies of rats without intercrossing for 12 or more generations justifies the designation of substrains. In our case the gap is some 15–25 generations. We know that our rats carry the same histocompatibility phenotype ($Hl^d$, E. Günther, pers. comm.) as the parent strain, but other differences may have emerged during the period of separate breeding. Experiments are on the way to screen for potential differences.

3 May 1978

M. ZÖLLER, S. MATZKU
Institute of Nuclear Medicine,
German Cancer Research Centre,
Heidelberg, FRG.